# Rapid, Inexpensive Measurement of Synthetic Bacterial Community Composition by Sanger Sequencing of Amplicon Mixtures

**DOI:** 10.1016/j.isci.2020.100915

**Published:** 2020-02-14

**Authors:** Nathan Cermak, Manoshi Sen Datta, Arolyn Conwill

**Affiliations:** 1 Fremont, CA, USA, 94555; 2 Department of Biology, Technion - Israel Institute of Technology, Haifa, Israel; 3 Physics of Living Systems, Department of Physics, Massachusetts Institute of Technology, Cambridge, MA 02139, USA; 4 Institute for Medical Engineering and Science, Massachusetts Institute of Technology, Cambridge, MA 02139, USA

**Keywords:** Microbiology, Microbial Genetics, Bioinformatics, Sequence Analysis

## Abstract

Synthetic bacterial communities are powerful tools for studying microbial ecology and evolution, as they enable rapid iteration between controlled laboratory experiments and theoretical modeling. However, their utility is hampered by the lack of fast, inexpensive, and accurate methods for quantifying bacterial community composition. Although next-generation amplicon sequencing can be very accurate, high costs (>$30 per sample) and turnaround times (>1 month) limit the nature and pace of experiments. Here, we quantify amplicon composition in synthetic bacterial communities through Sanger sequencing. We PCR amplify a universal marker gene, then we sequence this amplicon mixture in a single Sanger sequencing reaction. We then fit the “mixed” electropherogram with contributions from each community member as a linear combination of time-warped single-strain electropherograms, allowing us to estimate the fractional amplicon abundance of each strain within the community. This approach can provide results within one day and costs ∼$5 per sample.

## Highlights

• Our method “CASEU” estimates amplicon composition from mixed Sanger electropherograms
• CASEU uses a marker gene and is fast (1-day turnaround) and inexpensive ($5/sample)
• CASEU is accurate to ±1% abundance in mock microbial communities with up to seven strains
• CASEU is available as a free, open-source R package

## Graphical Abstract

**Figure:**
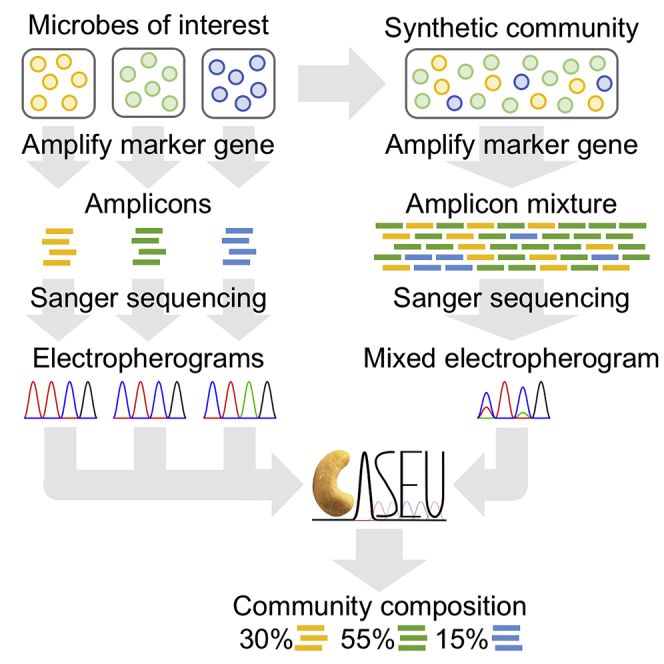

## Introduction

Model microbial communities, comprising a small number of pre-defined, culturable taxa, are emerging as powerful tools in microbial ecology and biotechnology. Unlike wild microbial communities, whose underlying design principles are often obscured by complex environmental conditions and thousands of microbial “parts,” simple synthetic consortia can be studied precisely under controlled laboratory conditions. Through this approach, numerous studies have uncovered principles of microbial community interactions, assembly, organization, and evolution (Celiker and Gore, 2014, Friedman et al., 2017, Goldford et al., 2018, Harcombe et al., 2014, Momeni et al., 2011, Momeni et al., 2017, Ratzke and Gore, 2018, Wolfe et al., 2014). Furthermore, simple synthetic consortia hold great promise for biotechnology (Brenner et al., 2008), including synthesis of natural products that would be difficult to achieve with a single species (Zhou et al., 2015).

Despite the importance of model microbial communities, characterizing their composition (the proportional abundances of their constituent strains) quickly and cheaply remains challenging, since most standard methods have significant drawbacks (Table 1). On the one hand, counting individual cells through colony formation on agar plates or with fluorescent labeling and flow cytometry is both cost- and time-effective and provides a direct measurement of population size. However, these methods can be applied only when strains are morphologically distinct or genetically tractable. On the other hand, next-generation sequencing can provide precise abundance estimates for arbitrary microbial communities, regardless of their composition, but typically has large up-front costs and can take weeks to months to receive results. Notably, all DNA-based methods provide estimates of gene or amplicon abundances, which are distinct from cell abundances because strains differ in their gene (Větrovský and Baldrian, 2013) and genome copy number (Akerlund et al., 1995, Schaechter et al., 1958), as well as extraction (Abusleme et al., 2014, Yuan et al., 2012) and amplification efficiency (Polz and Cavanaugh, 1998).

**Table 1:** Comparison of Methods for Determining Strain Composition in Simple Model Microbial Communities

Method	Cost Considerations	Speed	Measurement Uncertainty	Biological Limitations
Illumina sequencing of marker gene amplicons	Entirely outsourced >$20/sample for library prep and sequencing^a^ In house >$1,500/lane at university facility, plus library prep costs ($37.5/sample)^b^	Typically weeks, sometimes months to get results^c^	Ideally limited by Poisson (counting) error. Given 50,000 reads, can detect members with abundance <0.01%	Requires marker gene that has unique sequence but conserved primer sites for all strains (e.g., 16S rRNA gene)
qPCR of marker genes	<$1/sample for PCR	Same day	Large dynamic range but low accuracy	Similar to Illumina sequencing. Requires designing specific primers or probes for each strain
Plate counts of CFUs	Low (requires only agar plates)	Typically 2–3 days, depending on growth rates	Ideally limited by Poisson error	Strains must produce morphologically distinct colonies. Communities must be dissociable to single cells
Fluorescent labeling of cells	Flow cytometer or microscope use	Same day	Ideally limited by Poisson error	Requires genetically tractable strains and spectrally distinct labels for each strain, potentially limiting communities to a few strains
CASEU (this work)	$4–6/sample for sequencing, plus <$1/sample for PCR	As fast as next day	Fractional abundance error typically 1 percentage point	Similar to Illumina sequencing for smaller consortia

Sanger sequencing prices and turnaround times were obtained from Genewiz (https://www.genewiz.com/Public/Services/Sanger-Sequencing/Purified-Templates, accessed 2018 Apr 10).

a CGEB—Integrated Microbiome Resource (http://cgeb-imr.ca/pricing.html., accessed 2018 Feb 17).

b BioMicroCenter:Pricing—OpenWetWare. (https://openwetware.org/wiki/BioMicroCenter:Pricing, accessed 2018 Feb 17).

c CGEB—Integrated Microbiome Resource (http://cgeb-imr.ca/queue.html, accessed 2018 Feb 17).

Sanger sequencing has long been a cheap and effective method to characterize the taxonomy of bacterial strains in isolation, often by sequencing the 16S rRNA gene. This process typically begins by PCR-amplifying the 16S rRNA gene(s) from a pure bacterial culture containing a single strain. The result is a homogeneous pool of 16S rRNA amplicons (unless the strain has multiple copies of the 16S rRNA gene). Subsequently, the amplicon pool is subjected to a linear amplification process that yields DNA segments of different lengths (Sanger et al., 1977), where all segments of a given length have a fluorescent color label corresponding to the final (3′) base (Smith et al., 1986). Then, DNA segments are sorted by length via capillary electrophoresis (Swerdlow and Gesteland, 1990), and the nucleotide sequence is determined from the corresponding sequence of fluorescent colors. Data are produced in the form of an electropherogram, in which fluorescent signal is plotted as a function of electrophoretic time (roughly corresponding to sequence position). Once characterized, the 16S rRNA gene sequence is often used as a taxonomic marker for a bacterial isolate.

In multi-strain bacterial communities where each member has a distinct 16S rRNA sequence, Sanger sequencing can be extended to characterize the presence and/or fractional abundance of each community member. The full complement of 16S rRNA genes present within a multi-strain community can also be PCR-amplified (typically with degenerate universal primers) and analyzed via Sanger sequencing. This process results in a “mixed” electropherogram. Like the single-strain electropherogram, a mixed electropherogram records the fluorescent signal as a function of electrophoretic time, but it now includes contributions from each of the strains present. Two approaches to characterize multi-strain community composition from mixed electropherograms have been developed previously (described below). However, unlike the new method we propose here, both prior approaches sought to characterize community composition without any prior knowledge of which strains were present.

In the first method (Kommedal et al., 2008), a novel base-calling method was developed to preserve ambiguity at positions where multiple nucleotides were present, thereby allowing the authors to enumerate every possible constituent sequence. They then compared possible sequences with a database of known 16S rRNA gene sequences. Using this method, they reliably identified the bacteria present in numerous two- and three-species mixtures, including clinical samples (Kommedal et al., 2009, Kommedal et al., 2011, Wolff et al., 2013). However, this approach has not been used for quantification of strain abundance, and it is unclear how accurately the members of more complex communities (>3 strains) can be resolved.

In the second method (Amir and Zuk, 2011), the authors developed an algorithm to find a sparse set of strains whose combined DNA would be expected to generate the observed mixed electropherogram. To do this, they first created a database of predicted electropherograms (based on a statistical model of how gene sequences determine electropherograms) for 16S rRNA sequences of nearly 20,000 bacterial strains. They then computationally solved for a small set of strains that could best reproduce the observed electropherogram. Applying this method to a mixture of five equally abundant strains, they detected at least eight strains, of which seven were closely related to strains in the actual mixture. However, their fractional abundance estimates were noisy, varying from 5%–15% when the actual abundances were 20% each.

Here we develop and evaluate a new and distinct method for analyzing Sanger sequencing traces from amplicon mixtures as a fast (1 day) and inexpensive (∼$5/sample) method for quantifying the fractional abundance of individual strains within simple model communities. It differs from previous approaches in two main ways. First, it assumes that one knows the full set of strains that might be in the mixture and experimentally measures their individual Sanger electropherograms. For model systems consisting of cultured isolates, this requirement is easily fulfilled. Second, our method accounts for a common mode of run-to-run variability not previously accounted for, which we show is necessary for accurate compositional estimates. We benchmark this method with multiple 2-, 4-, and 7-member communities of marine bacterial isolates, achieving a root-mean-square error of roughly 1% and yielding results similar to Illumina sequencing. We also demonstrate the utility of this method by quantifying time dynamics of five model communities over 2 weeks. Overall, given its accuracy and broad applicability, we believe that this method will enable experiments with a wide range of simple synthetic microbial communities that were previously time- or cost-prohibitive.

We have also implemented our method in a free and open-source package for the open-source language R (R Core Team, 2017, Hill et al., 2014) under the name “CASEU” for Community/Compositional Analysis via Sanger Electropherogram Unmixing. We provide functions for fitting and evaluating fit quality, both via the R language/terminal and through a graphical user interface.

### Approach

Our approach is to fit mixed-strain electropherograms as linear combinations of time-warped single-strain electropherograms. For a model bacterial community in which all component strains are known, it is possible to measure its mixed electropherogram, as well as each single-strain electropherogram. We thus sought to find a function relating the two that would allow us to extract the relative proportions of individual strains in the mixed electropherogram.

#### A Simple Linear Model Is Insufficient due to Retention-Time Variability

Naively, it is reasonable to fit a mixed Sanger electropherogram as an abundance-weighted linear combination of single-strain electropherograms. However, this approach yields poor fits owing to between-sample and within-sample variability in the run speed, that is, the rate at which molecules migrate during electrophoresis. This phenomenon, referred to as “retention-time variability,” is a well-known confounding factor in electrophoretic methods (Eilers, 2004, Nielsen et al., 1998), including Sanger sequencing. Indeed, we observed substantial retention-time variability in our measurements: technical replicates of the same sample sequenced on different days were often temporally offset from each other (by roughly ±1 base) and were sometimes stretched or contracted relative to one another by ±0.3% (see Figures S1 and S2).

**Figure 1:**
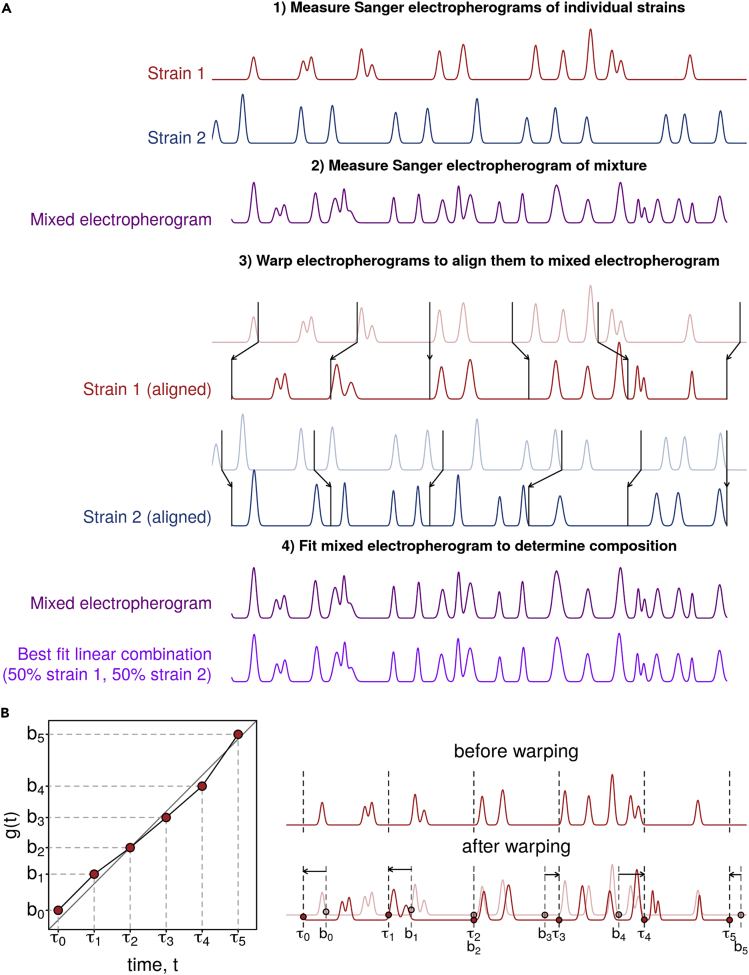
CASEU Quantifies the Fraction of Individual Strains in Mixed Communities by Fitting Mixed Sanger Electropherograms as Linear Combinations Of Time-Warped Single-Strain Electropherograms (A) Schematic of CASEU approach. Electropherograms shown are simulated for illustration purposes. For clarity, only a single fluorescence channel is illustrated. (B) Example of the continuous piecewise warping function used for alignment. The warping function is parameterized by six numbers, *b0*-*b5* (the values of the function at τ0, τ1, …, τ5). The figure shows an exaggerated warping with simulated electropherograms for illustration purposes.

Instead, our fitting procedure conceptually involves two components: time warping, which accounts for retention-time variability and fitting a linear model. First, we warp (locally shift and stretch or contract) the time axis of single-strain electropherograms (Figure 1). Second, we estimate strain abundances by fitting the mixed electropherogram as a linear combination of time-warped single-strain electropherograms. In practice, we do these steps simultaneously, by identifying warping parameters and abundance fractions that minimize the sum-of-squares difference between the observed and model-predicted mixed electropherogram, as follows:

(Equation 1) $${\text{argmin}}_{f_{1},\ldots ,f_{n},g_{1}\left(t\right),\ldots ,g_{n}\left(t\right),\;x_{0}}\overset{t_{end}}{\underset{t=t_{0}}{\sum }}\overset{4}{\underset{c=1}{\sum }}{\left(Y\left[t,c\right]-\left(x_{0}+\sum_{i=1}^{n}f_{i}X_{i}\left[g_{i}\left(t\right),c\right]\right)\right)}^{2}\;\;+\;\lambda \sum_{i=1}^{n}R\left(g_{i}\right)\;$$

where

• *f*_*i*_ is the abundance of strain *i*, where *i* ranges from 1 to *n*;
• *t* is an index of time, ranging from *t*_0_ to *t*_*end*_;
• *Y*[*t*,*c*] is a matrix of the mixed electropherogram, with one row per time point and one column for each of the four fluorescence channels, *c*;
• *X*_*i*_[*t*,*c*] is a matrix of strain *i*'s electropherogram, with one row per time point and one column for each of the four fluorescence channels, *c*;
• *x*_0_ is a scalar accounting for constant background fluorescence;
• *g*_*i*_(*t*) is a warping function for strain *i*; and
• *R*(*g*_*i*_) is a quadratic penalty function for shifting and stretching individual electropherograms.

Because it is not possible to have negative abundances, we constrain strain abundances to be non-negative (*f*_*i*_ > 0) by using non-negative least-squares fitting (Lawson and Hanson, 1995).

#### A Piecewise-Linear Time-Warping Function Can Account for Retention-Time Variability

Within a given electropherogram, the relative run “speed” may vary substantially, such that certain sections are stretched and others are contracted, compared with the average speed. To account for within-electropherogram variability, we use a continuous piecewise linear warping function *g*_*i*_(*t*) (see Figure 1B), which divides the electropherogram into several segments, each of which can be locally stretched or contracted (Nielsen et al., 1998). To prevent unreasonably large stretching or shifting, our software package includes the option of a quadratic penalty for moving the end of each segment from its original location ($R\left(g\right)=\;\underset{j}{\sum }{\left(\tau_{j}-b_{j}\right)}^{2}$ where *τ*_*j*_ and *b*_*j*_ are as shown in Figure 1B). However, for our data analysis in this work, we did not use this regularization term (*λ* = 0). To determine the optimal number of segments, we systematically varied the number of segments and aligned technical replicates to each other. We found that using five segments enabled us to align all samples precisely to either of their two technical replicates over a region of ∼630 bases (Figure S2). Using only a single segment yielded poor alignments between technical replicates (Figure S2) and produced mediocre estimates of known mixture fractions (Figure S3). Using more than five segments did not improve the alignments between technical replicates (Figure S2) but increased computation time.

## Results

To benchmark CASEU's performance, we analyzed a series of mock bacterial communities of 2, 4, or 7 bacterial strains with known fractional abundances. We prepared these communities by PCR-amplifying the 16S rRNA gene from each single strain (here called “A” through “H”) and mixing together amplicons from different strains in known fractions. By analyzing mixtures of amplicons, rather than mixtures of cells, we could measure sequencing and algorithmic performance independent of biases due to DNA extraction efficiency, PCR efficiency, or 16S rRNA gene copy number. Using mock communities, we assessed the following metrics of algorithmic performance:

• Accuracy of fractional abundance estimates, by systematically varying the abundance of community members between 1.3% and 95%;
• Reproducibility, by sequencing each sample three times on separate days;
• Ability to differentiate closely related strains, by varying phylogenetic distance between strains; and
• Ability to correctly reject the presence of “decoy strains,” which are included as potential community members in the fits but were absent in reality.

**Figure 2:**
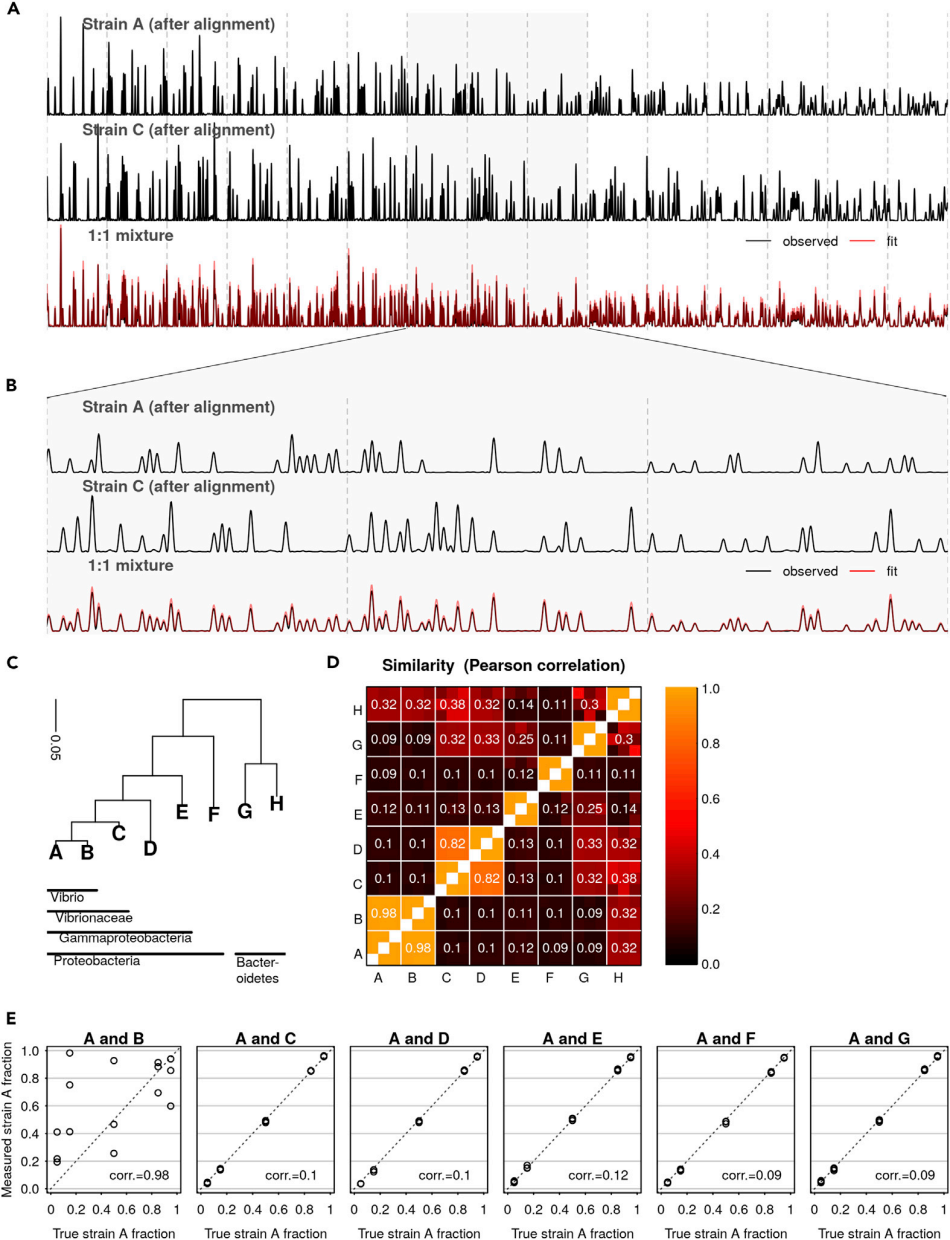
CASEU Accurately Resolves Composition of Two-Strain Mock Communities (A) An example alignment and fit over approximately 630 bases, showing a single fluorescence channel. Top and middle traces show reference electropherograms of individual strains (after warping). Bottom trace shows the electropherogram of a 1:1 mixture (black) and best-fit weighted sum of aligned references (red). (B) Zoom-in of segment of (A), showing alignments and fit over approximately 120 bases. (C) Phylogenetic tree of genes chosen for analysis, made using nearly full-length 16S sequences from Datta et al. (2016). We aligned these sequences using the SINA Alignment Server (Pruesse et al., 2012) https://www.arb-silva.de/aligner/ and made an approximate maximum-likelihood tree using FastTree 2.1.10 with the default options (Price et al., 2010). (D) Similarity matrix between all strains used in Figures 2, 3, and 4. Similarity was calculated as the Pearson correlation between electropherograms after aligning one to the other. Because each strain was measured in triplicate, each strain pair consists of a 3 × 3 submatrix of similarity values. The average of these replicate pairs is written in the figure. (E) Estimated mixture fractions plotted against the true ratio at which the sequences were mixed (circles). We also note the similarity (Pearson correlation) between strains. These mixture fractions have been corrected for errors in stock concentration (uncorrected fraction data shown in Figure S3, bottom row).

We first analyzed two-strain mixtures for which the proportion of a single strain varied from 5% to 95% (Figure 2). Across all two-strain communities (except the mixtures of strains A and B, see below), fractional abundance estimates were accurate with an average absolute deviation between the expected fraction and the observed fraction of 0.9 percentage points (range 0.05%–3%). Furthermore, abundance estimates were consistent across independently sequenced technical replicates; the average standard deviation of triplicate measurements was 0.59 percentage points (range 0.06%–1.17%).

**Figure 3:**
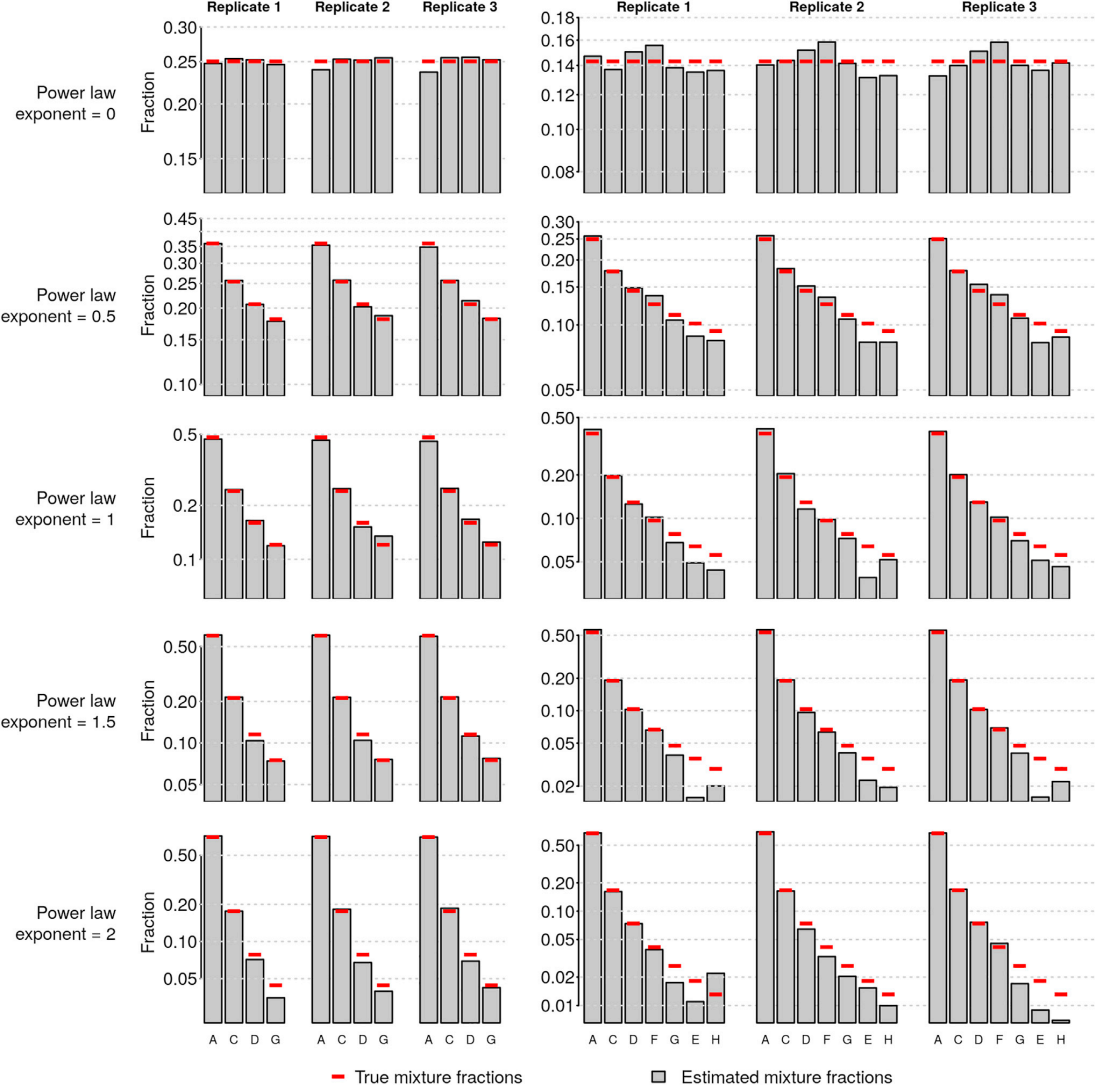
CASEU Provides Reliable Estimates of Community Composition in Mixtures of 16S Amplicons from Four (Left) or Seven (Right) Strains Solid bars show measurements after accounting for stock concentration error (uncorrected data are given in Figure S6); red lines show true mixture proportions based on power law distributions. In power law distributions, the abundance of the ith most abundant strain is proportional to $\frac{1}{i^{\alpha }}$ where α is the power law exponent.

In larger communities (4- and 7-strain mixtures), fractional abundance estimates were similarly accurate, even for low-abundance community members. To test the effect of strain evenness on fractional abundance estimates, we prepared 4- or 7-strain communities whose strain abundances were distributed according to a power law (*f*_*i*_∝*i*^−*α*^), where we varied the value of the exponent *α*. This allowed us to assemble communities of varying evenness (Figure 3), ranging from those in which all strains were at equal abundance (*α* = 0) to those in which the dominant strain was 50-fold more abundant than the least abundant strain (*α* = 2). Across these communities, abundance estimates were similarly accurate compared with the two-strain communities, with root-mean-square (RMS) errors of 0.75 and 1.14 percentage points (maximum errors of 2.2 and 3.4 percentage points), respectively, for the 4- and 7-strain communities. Furthermore, the magnitude of error in a strain's abundance was nearly independent of that strain's abundance in the community (Figure S5A). The standard deviations we observe between triplicate results were comparable with what would be attained by counting-based methods (e.g., next-generation sequencing or plate counts) with ∼5,000 counts (reads or colonies) per sample (Figure S5B).

**Figure 4:**
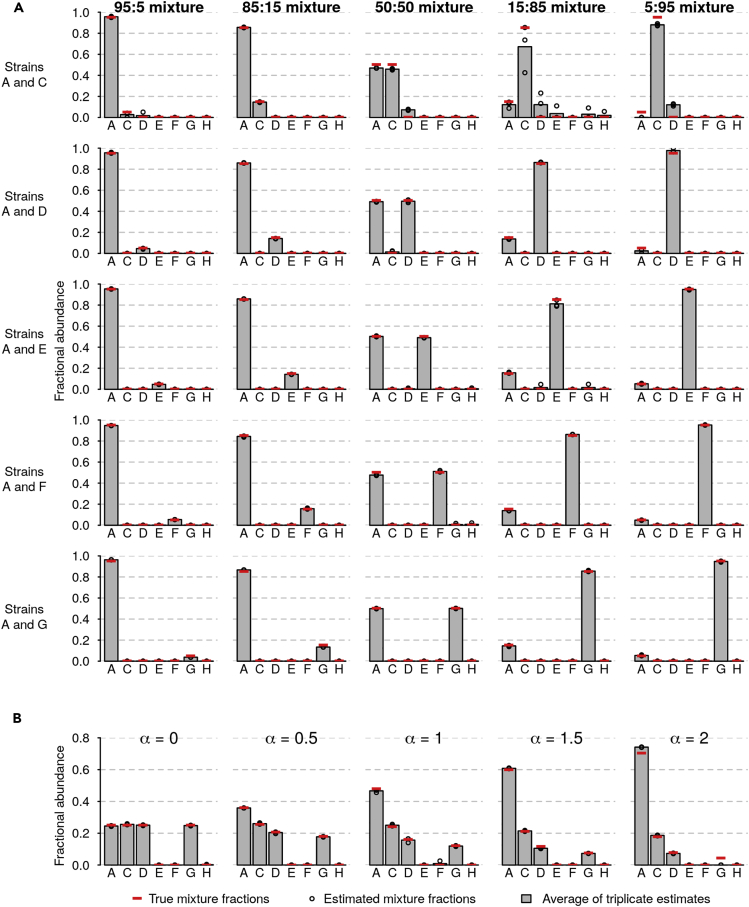
CASEU Correctly Infers the Absence of Strains that Were Not Present in the Mixture (A) Community composition estimates of two-strain mixtures (as in Figure 2D), in which five extra “decoy” strains were included as potential community members to test CASEU's ability to infer strain absence. Bars indicate CASEU estimates (average of three replicates); open circles indicate each of the three replicate estimates, and red lines indicate the true values. Estimates are corrected for errors in stock concentrations. (B) Community composition estimates of four-strain mixtures (as in Figure 3; α is the power law exponent), in which three extra “decoy” strains were included as potential members. Bars and points are as in (A).

It is important to not only estimate the abundance of a strain known to be present, but also to correctly determine when a strain is absent. To test whether CASEU is susceptible to erroneously finding strains that were not present, we re-fit all our two- and four-species communities, this time including all strains (except for B) as possible “decoy” community members. In nearly all cases, CASEU correctly rejected the presence of strains that were not included in the community (Figure 4). Notably, CASEU erroneously found non-zero amounts of strain D in some samples where only strains A and C were present. We attribute this to the similarity between electropherograms of strains C and D (discussed below).

### To Differentiate Strains, CASEU Requires That Their Electropherograms Are Dissimilar

We quantified similarity as the correlation between two electropherograms after aligning one to the other. Our mock communities contained mixtures of strains with varying degrees of electropherogram similarity, ranging from 0.98 (for strains A and B) to 0.09 (for strains A and G) over a 630-basepair region of the 16S rRNA gene (Figure S4). Although CASEU failed to differentiate strains A and B, which have an average post-alignment correlation of 0.98 (Figure 2), it accurately estimated fractional abundances for all other communities (Figures 2 and 3) containing between-strain correlations of up to 0.82 (strains C and D; Figure 3). However, strain D was sometimes mistakenly found in the mixtures of strains A and C, suggesting it may sometimes be mistaken for strain C. Therefore, we suggest that strains with correlations of ∼0.8 or greater may not be clearly resolvable with CASEU and should be analyzed with caution. In our dataset, this corresponds to roughly within-genus distances or closer, but the relationship between CASEU resolvability and phylogeny may depend on the specific strains of interest.

We also note that we expect electropherogram correlations to be strongly affected by indels, because our alignment approach has insufficient flexibility to accommodate large gaps. Strains A and B differ by only SNPs, whereas strain C possesses a 12-base deletion near the beginning of the gene (Table S1). This likely explains the low correlation and subsequent ability to differentiate A and C (which otherwise have only 29 SNPs in their 930 bases of high-quality sequence) but not A and B (which have no indels and only a dozen SNPs in one gene region).

We next investigated whether we could improve our results for the mixture of strains A and B by focusing on the region in which these two strains' electropherograms differ. Our logic was that if most of the electropherogram is uninformative and subject to some amplitude noise, then removing the uninformative regions should improve the signal-to-noise ratio. We thus fit only a small region of the electropherogram (roughly 59 bp) that included the differing bases between strains A and B (Figure S4). This enabled us to obtain far more accurate fractional abundance estimates for mixtures of strains A and B (Figure S4D). We thus suggest that CASEU users seeking to differentiate highly similar strains restrict their analysis to the region in which their electropherograms vary.

### Evaluation on Synthetic Model Communities

**Figure 5:**
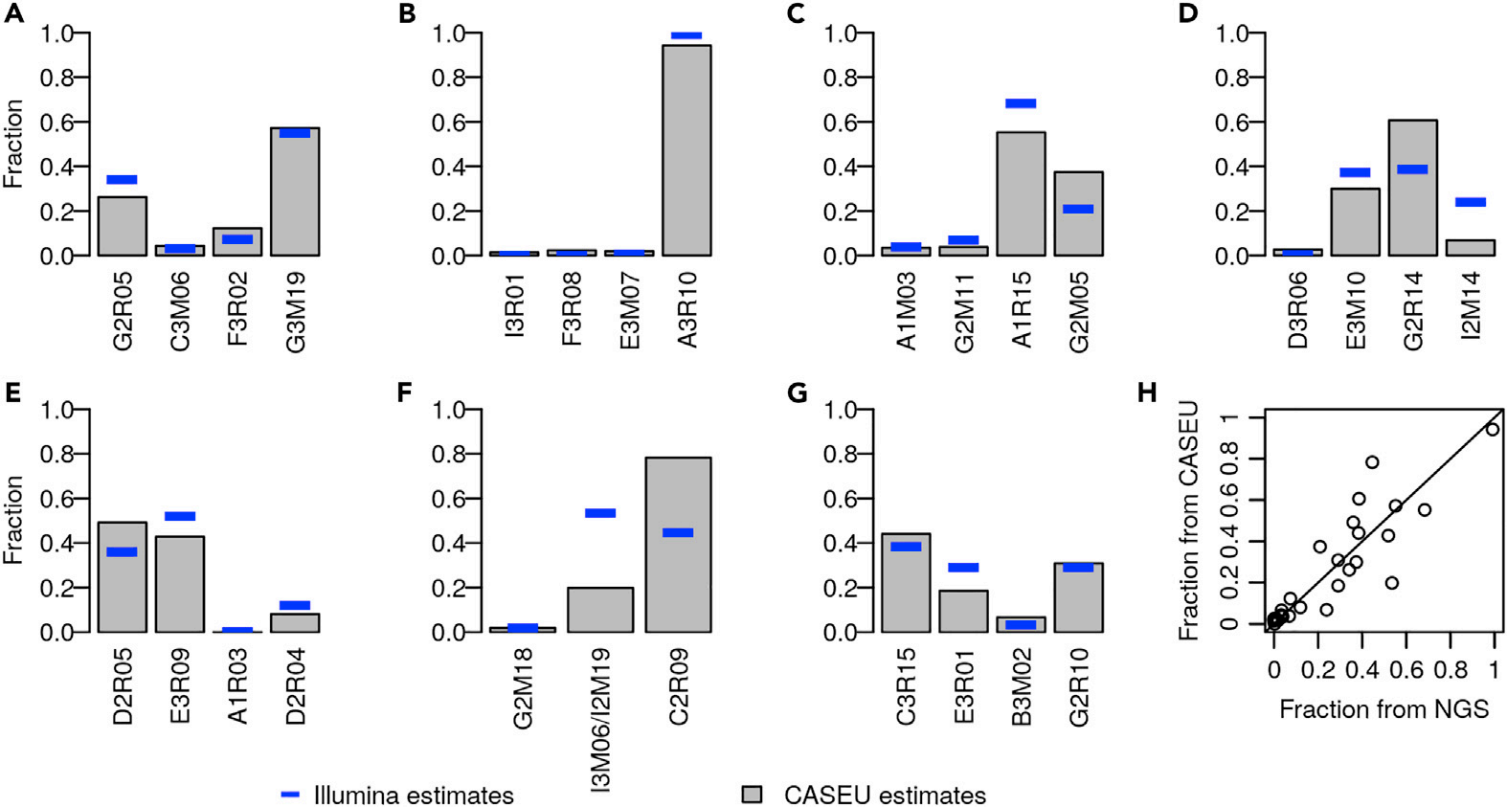
CASEU Yields Estimates of Community Composition That Are Typically Consistent with Illumina 16S Sequencing (A–G) Bar plots indicate results of CASEU analyses of mixtures of saturated cultures of four bacterial strains. Solid blue lines show estimates obtained from Illumina sequencing of the 16S rRNA V4-V5 hypervariable region. (H) Fractional abundance estimates for CASEU versus Illumina sequencing. Solid line shows equality.

We envision CASEU as a rapid, inexpensive alternative to Illumina sequencing for characterizing the structure of simple synthetic microbial communities. To demonstrate this use case, we performed experiments with seven four-strain model communities of unknown fractional composition, derived from strains isolated in Enke et al. (2019). We extracted DNA from each community, then amplified and sequenced each sample twice, once via Sanger sequencing (16S rRNA V1-V9 hypervariable regions) and once via next-generation Illumina sequencing (16S rRNA V4-V5 hypervariable regions) (Figure 5). Importantly, this analysis does not compare the accuracy of the two methods, since the true fractional abundances are unknown, but rather whether their fractional abundance estimates are consistent.

We found that CASEU provided community composition estimates consistent with next-generation Illumina sequencing of 16S rRNA amplicons (Figure 5), despite differences in library preparation procedure and sequencing technology. Across all communities, fractional abundance estimates between the two methods were highly correlated (Pearson correlation 0.88, Figure 5H). Furthermore, in five of seven communities, we observed strong quantitative agreement between Illumina estimates and CASEU estimates, with an RMS difference of 7.0 percentage points (Figure 5A–5C, 5E, and 5G).

In the two model communities where CASEU and Illumina sequencing disagreed (Figures 5D and 5F; RMS differences of 15 and 28 percentage points, respectively), the differences can be attributed to a single group of closely related strains. These three strains (I3M06, I2M14, and I2M19) are very closely related (electropherograms cannot be distinguished by CASEU), and in both model communities, these strains were estimated by CASEU to be at substantially lower fractions than was estimated by Illumina sequencing. Although we remain uncertain as to why these strains are detected less with CASEU than Illumina, it may be a result of CASEU and Illumina relying on different primers and amplification protocols.

In our CASEU analyses performed with these four-strain model communities, we additionally observed two cases in which CASEU produced poor fits as quantified by the correlation between the observed and predicted traces (Figure S7). In the first case, the predicted electropherogram had a correlation of only 0.63 to the observed electropherogram, compared with >0.9 for all other samples. This poor fit alerted us to a low-quality Sanger sequencing electropherogram for one strain in the community, which contained a large anomalous fluorescence spike (Figure S7A). In the second case, CASEU yielded a correlation between predicted and observed traces of 0.45, compared with >0.95 for other samples from the same model community. This poor fit was caused by the presence of a contaminating strain, which was not included in the fit (Figure S7B). Including the contaminating strain increased the fit correlation to 0.95. Thus, while we only rarely observed poor fits, CASEU includes a simple metric that enables users to identify and exclude problematic samples.

## Discussion

In microbial ecology, model communities have emerged as a useful intermediate between single-species microbiology and complex natural communities. Here, we demonstrate that Sanger sequencing can be used for rapid, inexpensive, and accurate quantification of model community composition.

### CASEU Can Provide Rapid Results

Sanger sequencing requires a simple sample preparation protocol with a single PCR step, followed by outsourced Sanger sequencing. Therefore, the time to acquire results is largely limited by sequencing time, which is often less than 1 day. In contrast, next-generation sequencing requires a more time-consuming library preparation protocol, often with multiple PCR steps for adaptor ligation and barcoding. Furthermore, runtime for an Illumina MiSeq routinely exceeds 1 day (e.g., 40 h for paired-end 150 × 150 sequencing) but can require weeks to months if outsourced.

### CASEU Can be Inexpensive

Sanger sequencing has a fixed cost per sample (here, $4 for sequencing and roughly $1 for PCR and cleanup), whereas Illumina sequencing has large upfront cost (typically more than $1,000 per sequencing lane), plus per-sample costs for library preparation.

### CASEU Is Accurate for Simple Model Communities

Sanger sequencing provides an accurate and reproducible means to quantify amplicon composition for model communities, achieving similar results as Illumina sequencing for model communities and errors of less than 1% point for mock communities.

More broadly, we believe that our Sanger sequencing demixing approach can be extended beyond the 16S gene. For example, CASEU might be used with model communities containing closely related strains by using other marker genes (e.g., Vibrio communities that are poorly resolved by 16S but easily differentiated by *hsp60* sequences [Hunt et al., 2008]), or even communities containing both fungal and bacterial members (for example, cheese rind model communities [Wolfe et al., 2014]) by amplifying both 16S and 18S or ITS sequences simultaneously through multiplexed PCR. Beyond microbes, CASEU might be extended to quantify aneuploidy using marker sequences with conserved primer sites present on all chromosomes (Kinde et al., 2012). Overall, we believe CASEU provides a versatile tool to assess sequence-variant composition in multiple contexts.

### Limitations of the Study

CASEU has important limitations. To determine if CASEU is appropriate for your application, we recommend considering the following factors as they pertain to your model community.

#### Number of Strains

Here, we demonstrate that CASEU can provide accurate fractional abundance estimates for communities of 2, 4, and 7 strains. However, CASEU may be suitable for larger model communities, as we did not identify an upper bound on the number of resolvable members.

#### Resolvability of Strains

We found that strain resolvability depends on the correlation of their electropherograms, which is distinct from their aligned sequence similarity. Therefore, for your particular community, we recommend Sanger sequencing each individual strain and verifying that their electropherograms cannot be aligned to be highly correlated, which can be done with our R package.

#### Low-Abundance Strains

Given typical errors of 1%–2%, CASEU cannot resolve community members at fractional abundances below 1%. If this dynamic range is needed, alternatives like qPCR or next-generation sequencing may be more suitable.

#### Sources of Bias

CASEU shares the same limitations of all DNA-based approaches for quantifying community composition, including bias in DNA extraction and amplification efficiency. Importantly, next-generation sequencing, qPCR, and CASEU do not yield cell counts but instead yield sequence abundance. Although sequence abundance is expected to be roughly proportional to cell count for any given strain, this relationship may vary between strains depending on gene copy number (Větrovský and Baldrian, 2013), growth phase (Akerlund et al., 1995, Hildenbrand et al., 2011, Schaechter et al., 1958), DNA extraction efficiency (e.g., Abusleme et al., 2014, Yuan et al., 2012), and amplification efficiency (e.g., Polz and Cavanaugh, 1998).

## Methods

All methods can be found in the accompanying Transparent Methods supplemental file.

## Data and Code Availability

We have implemented our method in a free and open-source R package called CASEU (“Community/Compositional Analysis by Sanger Electropherogram Unmixing”), available at https://bitbucket.org/DattaManoshi/caseu.

## Supplemental Information

Document S1. Transparent Methods, Figures S1–S7, and Table S1
