# Supplementary material for: Rapid, Inexpensive Measurement of Synthetic Bacterial Community Composition by Sanger Sequencing of Amplicon Mixtures
Source: iScience. 2020 Feb 14;23(3):100915. doi: 10.1016/j.isci.2020.100915 (PMC7047173; doi:10.1016/j.isci.2020.100915)
Supplement: Document S1. Transparent Methods, Figures S1–S7, and Table S1 [file mmc1.pdf]

iScience, Volume *23*

Supplemental Information

Rapid, Inexpensive Measurement of Synthetic Bacterial Community Composition by Sanger Sequencing of Amplicon Mixtures

Nathan Cermak, Manoshi Sen Datta, and Arolyn Conwill

Transparent Methods

Strains. Strains A-H are marine isolates collected in (Datta et al., 2016) from Canoe Beach, Nahant, MA, USA. The taxonomic identities of the isolates (classified using SINA (Pruesse et al., 2012)) are as follows: Strain A (1A01), Vibrio; Strain B (4B04), Vibrio; Strain C (6D03), Vibrionaceae; Strain D (6C06), Psychromonas; Strain E (4A09), Oceanospirillaceae; Strain F (4A10), Rhodobacteraceae; Strain G (4C08), Polaribacter; Strain H (6B07). Strains in Figs. 5 and S7 are isolates from (Enke et al., 2019), collected from the same location. Communities are denoted by their subfigure label in Fig. 5.

A: G2R05 Cellulophaga, C3M06 Rhodobacteraceae, F3R02 Neptunomonas, G3M19 Celeribacter
B: I3R01 Vibrio, F3R08 Shewanella, E3M07 Paraglaciecola, A3R10 Tenacibaculum
C: A1M03 Alteromonas, G2M11 Colwellia, A1R15 Pseudoalteromonas, G2M05 Photobacterium
D: D3R06 Colwellia, E3M10 Cellulophaga, G2R14 Vibrio, I2M14 Marinobacterium
E: D2R05 Alteromonadaceae, E3R09 Winogradskyella, A1R03 Shewanella, D2R04 Rhodobacteraceae
F: G2M18 Saccharospirillaceae, I3M06 Marinobacterium, C2R09 Paracoccus, I2M19 Marinobacterium
G: C3R15 Flavobacteriaceae, E3R01 Tenacibaculum, B3M02 Psychromonas, G2R10 Vibrio

Preparing mixtures of 16S amplicons and sequencing. For two-, four-, and seven-strain mixtures, genomic DNA was extracted as previously reported[15]. 16S genes were amplified with 27F (AGAGTTTGATCMTGGCTCAG) and 1492R (TACGGYTACCTTGTTACGACTT) universal primers, as follows:

Reagent	Volume
ddH2O	23.5µL
5X HF Buffer	10 µL
dNTPs (10mM)	1 µL
27F primer (3uM)	5 µL
1492R primer (3uM)	5 µL
Phusion polymerase	0.5 µL
Genomic DNA	5 µL
Total	50 µL

PCR cycle conditions were as follows:

Step	Temperature	Duration
Initial denaturation	98°C	30 seconds
Amplification (30 cycles)	98°C	30 seconds
	50°C	30 seconds
	72°C	90 seconds
Final extension	72°C	10 minutes

For experiments shown in Figs. 2 and 3, we ran six PCRs for each strain to ensure that we had sufficient amplicon DNA to prepare all the mixtures. We pooled each set of six reactions into a single tube, then SPRI-cleaned the products. We estimated DNA concentrations via Nanodrop,

and subsequently diluted all samples to 3 ng/μL (concentration measurements required subsequent computational correction, see Materials and Methods). We added 5 μL of 27F primer at 15 μM to 40 μL of amplicon at 3 ng/μL, to yield 45 μL with a primer concentration of 1.6 μM and a template concentration of 2.6 ng/μL. These concentrations are what Genewiz recommends for Sanger sequencing (https://www.genewiz.com/Public/Resources/Sample-Submission-Guidelines/Sanger-Sequencing-Sample-Submission-Guidelines/Sample-Preparation#sanger-sequence, accessed 2018 Apr 2). We split the 45 μL of sample into three separate plates, each with 15 μL of sample per well, and submitted each plate on a different day over the course of one week.

Sequencing was performed by Genewiz as a drop-off service for $6/sample (<48 samples) or $4/sample (>48 samples). We routinely received results within 24 hours of submitting our samples. Our ABIF file metadata suggests Genewiz sequencing was performed on a 3730xl DNA Analyzer, using BigDyeV3.

Processing ABIF files. We used the 'sangerseqR' Bioconductor package (Hill et al., 2014) in R (R Core Team, 2017) to read in ABIF (.ab1) files. In ABIF files, there are two types of data we considered using: "raw" fluorescence traces, and "processed" data. While the details of the processing method are not available, the process appears to involve baseline subtraction, low-pass filtering, and an unknown temporal adjustment. Attempts to use the "raw" traces were stymied by the poor temporal alignment of the traces and required searching a much larger range of alignment parameters, yielding a significantly slower analysis. Before analysis, we additionally normalized the amplitudes of all reference files such that the mean amplitude was one over the region to be used for alignment.

Algorithm for fitting mixed electropherograms. We initially tried optimizing Equation (1) via the Nelder-Mead algorithm (also called downhill simplex) but found that this method tended to yield solutions that were very dependent on starting estimates (suggesting many local minima). Instead, we adopted an approach in which we determine the warping parameters for one strain at a time. To determine the warping parameters for a single strain ("aligning a single strain"), we use the dynamic programming approach pioneered in correlation-optimized warping(Nielsen et al., 1998). In brief, we first calculate the sum of squared errors over a 2D grid of values for parameters b_1 and b_2 (the boundaries of the first warping segment). (Note that to do so, we rapidly calculate optimal f_i values for each (b_1, b_2) pair using non-negative least squares.) For each possible value of b_2, we only keep track of the best value of b_1 and its corresponding error. We then repeat that same process, but this time evaluating the error for a grid of possible values for b_2 and b_3 (the boundaries of the second segment). For every b_2-b_3 pairing, we calculate the error over that segment, plus the lowest possible error for that value of b_2 over any value of b_1. We then record the lowest error obtainable for any given value of b_3, and the corresponding b_2 that yields that optimum. We repeat this process for all five warping segments. This process does not necessarily yield a globally optimal solution because, for the errors for each segment to be additive, we allowed each segment to have its own amplitude parameter f_i. However, ultimately this parameter f_i, must be the same for all segments. We thus globally refine parameter estimates by minimizing equation (1) directly via Nelder-Mead, starting from the (b_1-b_6) estimates obtained as described above (which are usually very near the final optimum).

To align multiple strains, we sequentially align strains one after another. We first align each strain individually, identify the one that yields the greatest improvement in the fit, and fix that strain's alignment parameters (b_1-b_6). We then repeat that process for the remaining strains, always

greedily fixing the parameters of whichever strain yields the greatest improvement in the fit (reduction in squared error). Our rationale was that this would ensure that we always fit the majority component of the mixture before fitting the minority components.

More formally, our algorithm is as follows:

Inputs:

- $Y[t, c]$ - Mixed electropherogram matrix
- $X_1[t, c], X_2[t, c], \ldots, X_n[t, c]$ - Individual reference electropherogram matrices (n is number of strains)

Run:

1. Initialize $F = \emptyset$, the indices of strains for which the alignment parameters have been fixed.
2. Initialize $U = \{1, \ldots, n\}$, the indices of strains for which the alignment parameters have not yet been fixed.
3. Initialize A as an empty $n \times 6$ matrix for the alignment parameters.
4. While $U \neq \emptyset$
    1. For every strain index i in U, find a warping of X_i via dynamic programming that yields the greatest reduction in equation (1), conditional on all strains with fixed alignments X_f for $f \in F$.
    2. Identify which strain j, yields the most improvement to the fit.
    3. Fine-tune the alignment parameters of strain j by downhill simplex.
    4. Record strain j's alignment parameters in row j of matrix A.
    5. Add strain j to F, remove it from U.
5. Fine-tune all alignment parameters simultaneously by optimizing over A via downhill simplex, starting from A (the best individual alignments obtained in step 4).

On a HP laptop with an Intel Core i7-7500U CPU and 16 GB RAM, our fitting approach took on average 10.8 seconds for a two-strain mixture, and 138 seconds to fit a seven-strain mixture. Notably, the computation time is expected to be at worst quadratic in the number of strains that could potentially be in the mixture.

Accounting for concentration errors. Before mixing amplicons to make mock communities, we attempted to normalize all amplicon stock solutions to the same concentration, as measured by Nanodrop. However, the Nanodrop has limited precision, and as such we have fit a model to correct for remaining non-uniformity in stock solution concentration. For all 120 amplicon mixture samples in Figs. 2 and 3, we fit a model in which the amplicon concentration of each strain i was C_i times greater than expected. We then calculate the mixture fractions that would have resulted had the concentrations been equal.

$$f_{i,corrected} = \frac{\frac{f_{i,observed}}{C_i}}{\sum_{j=1}^{n} \frac{f_{j,observed}}{C_j}}$$

We estimated C_i values by finding the C_i that minimized the mean squared difference between the corrected fractions and the known fractions at which the amplicon stock solutions were volumetrically mixed. Arbitrarily, C_A was set to 1, and all C_i values were defined relative to that (therefore our model has seven free parameters). Concentration error estimates were as follows: C_B=5.35 (not reliable due to poor estimates of $f_{observed}$), C_C=1.10, C_D=0.80, C_E=0.66, C_F=0.91, C_G=0.86, C_H=0.75.

Model community dynamics and Illumina sequencing.
Communities used in Fig. 5 contained non-overlapping sets of four marine isolates. We grew communities at room temperature while shaking in 200uL of 2216 Marine Broth media in a 96-well deep well plate. Communities were diluted 100-fold and transferred to new plates every 24 hours and sampled after two weeks for DNA extraction and sequencing. DNA extraction was performed using a Epicentre MasterPure Kit.

Illumina 16S V4-V5 library preparation and sequencing were performed at Integrated Microbiome Resource (IMR) on an Illumina MiSeq (paired-end, 300-basepair reads). For Sanger sequencing, samples were PCR-amplified using identical conditions as described for the amplicon mixtures (above), but in 25 µL volumes instead of 50 µL.

Analysis of Illumina sequencing data. Illumina sequencing of model communities resulted in 514,524 reads (average per sample of 64,316 reads, range over all samples of 46,633-72,095 reads). Paired-end reads were merged with vsearch –fastq_mergepairs (10 mismatches allowed in overlap region) and trimmed of primer sequences with cutadapt 1.16. We estimated read counts for each isolate by assigning trimmed and merged reads that perfectly matched a known isolate 16S rRNA V4-V5 sequence to that isolate. Reads that did not match a known isolate were discarded. Fractional abundances were estimated by dividing the read count for each isolate in a sample by the read counts for all isolates in that sample.

Supplemental Figures and Tables

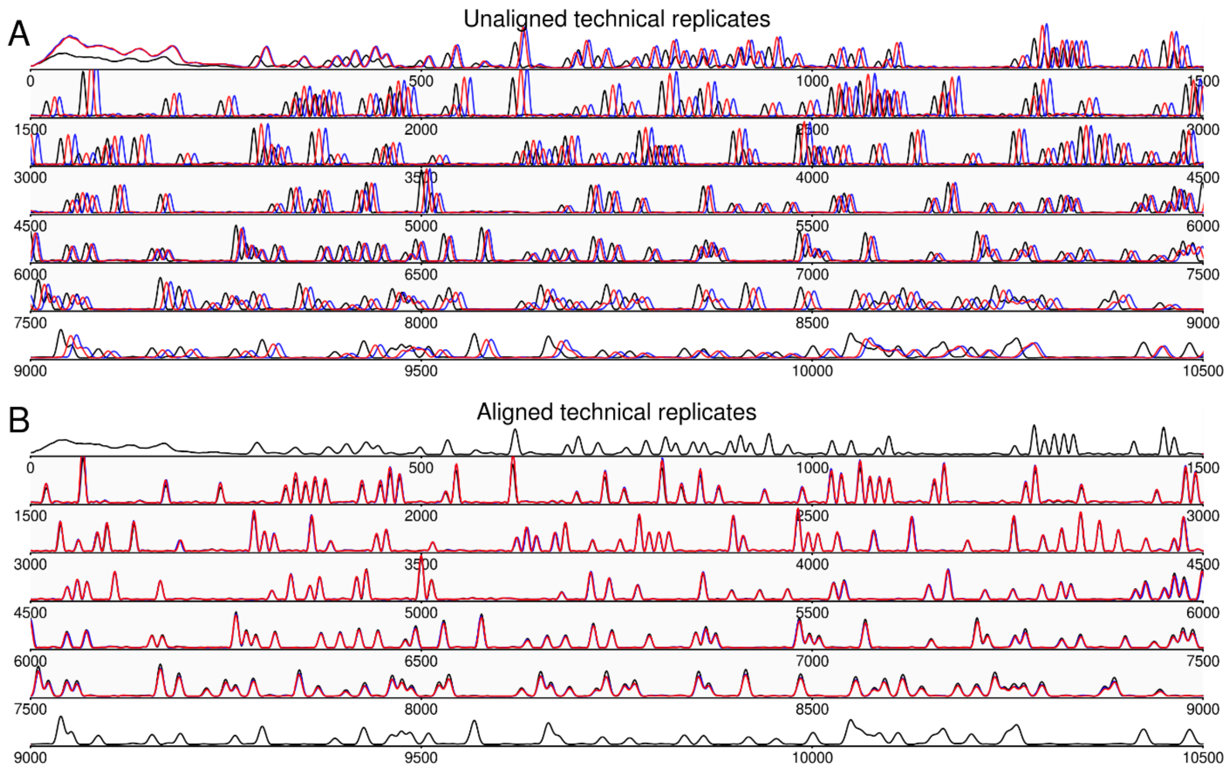

Fig S1. Aligning via time-warping can correct for temporal variability among technical replicates. Related to Figure 1**.**

(A) Three technical replicates (black, red and blue) for a sample of 16S DNA (showing only a single fluorescence channel for clarity).

(B) Technical replicates two and three aligned to technical replicate one, over indices 1500-9000, covering ~630 bases.

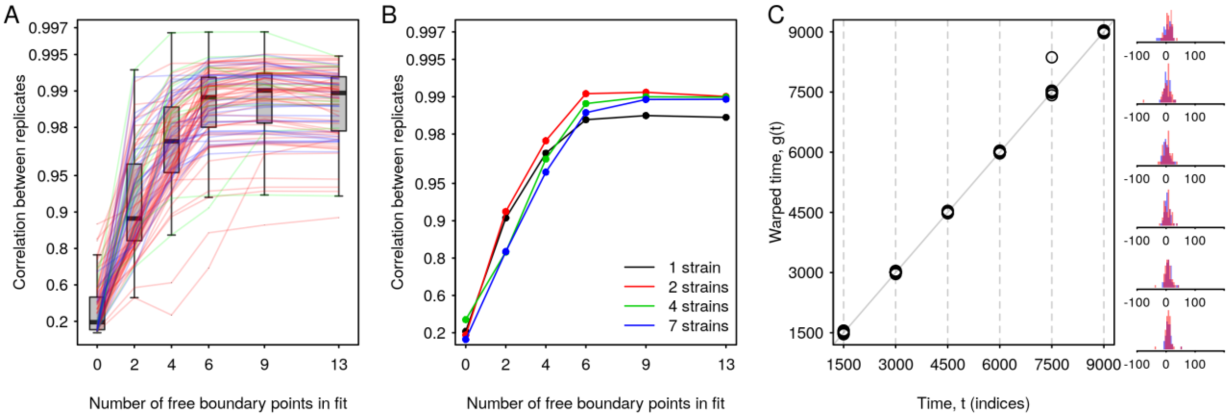

Fig S2. Determining optimal warping flexibility and typical range of warping parameters. Related to Figure 1.

(A) A six-parameter alignment yields good fits without unnecessary degrees of freedom. Fit quality was quantified as post-alignment Pearson correlation between technical replicates. With less than six boundary parameters, fits can be improved by increasing the warping flexibility, but beyond six parameters improvements are minimal. Lines indicate individual replicates and are colored according to the legend shown in (B).

(B) Same data as in (A) but averaged for each type of mixture. The similarity between lines shows that the required flexibility of the warping function does not depend on the sample type.

(C) Warping functions estimated from fitting each sample to its two technical replicates (N=48 samples; 8 single-strain samples; 30 two-strain samples; 5 four-strain samples; 5 seven-strain samples). At right are histograms of offsets for each boundary (how many time indices the boundary was moved in the alignment). Pink bars show alignment parameters for replicate 2, blue bars show alignment parameters for replicate 3, suggesting no day-specific effects.

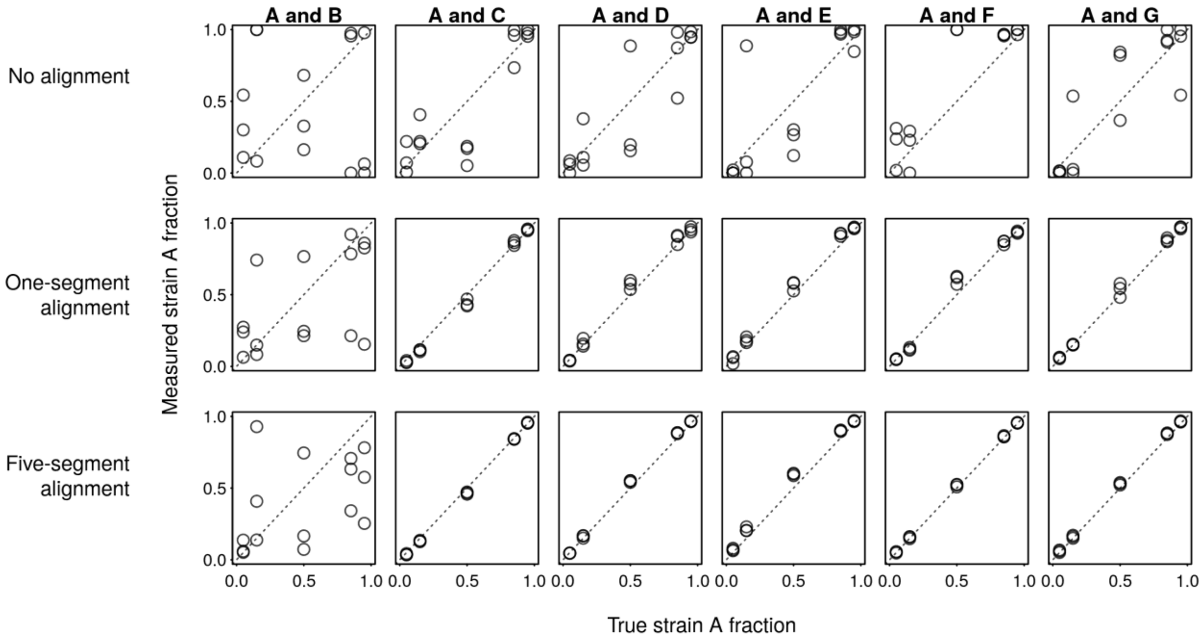

Fig S3. Flexible alignment is necessary to quantify community composition accurately. Related to figure 2. Without alignment, estimates of the fractions of each component are poor (top row). With 2-parameter alignment it yields more accurate results (middle row), but still much less precise than those obtained with 6-parameter alignment (bottom row). Data in this figure is not corrected for error in stock solution concentrations.

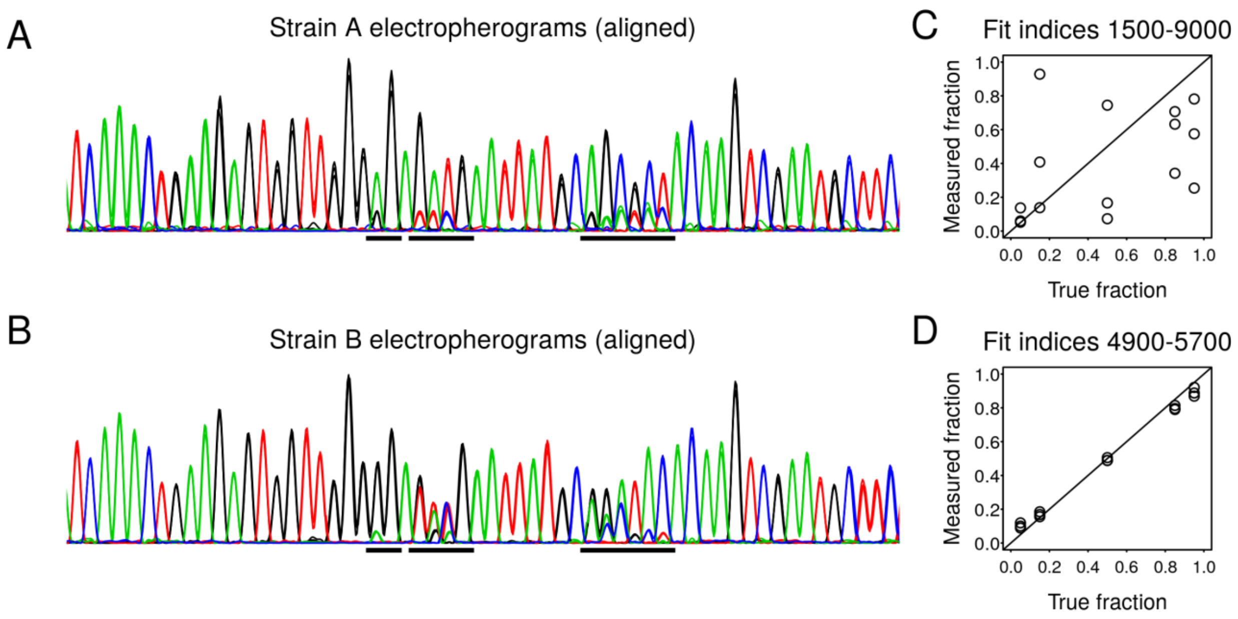

Fig S4. Strains A and B have similar electropherograms, but can be quantified by CASEU when restricting the analysis to the differing region. Related to figure 2.

(A) Overlay of aligned triplicate electropherograms of strain A, spanning electropherogram indices 4900-5700 (total electropherogram length is typically ~13000 indices, fit region is indices 1500-9000 for all other analyses in this paper). Colors correspond to the four fluorescence channels.

(B) Overlay of electropherograms of strain B, aligned to an arbitrarily-chosen replicate of strain A. Underlined stretches of ~6-7 bases are the positions at which these electropherograms differ. Over the remainder of the fit region, the electropherograms yield identical sequences.

(C) Measured fractional abundances of strain in mixtures of strains A and B (same as lower left panel of Fig S3), fitting the full electropherogram region as used throughout the rest of the paper, approximately 630 bases.

(D) Same as (C), but restricting the fitting to roughly the region in which the two genes differ (the region shown in (A) and (B)).

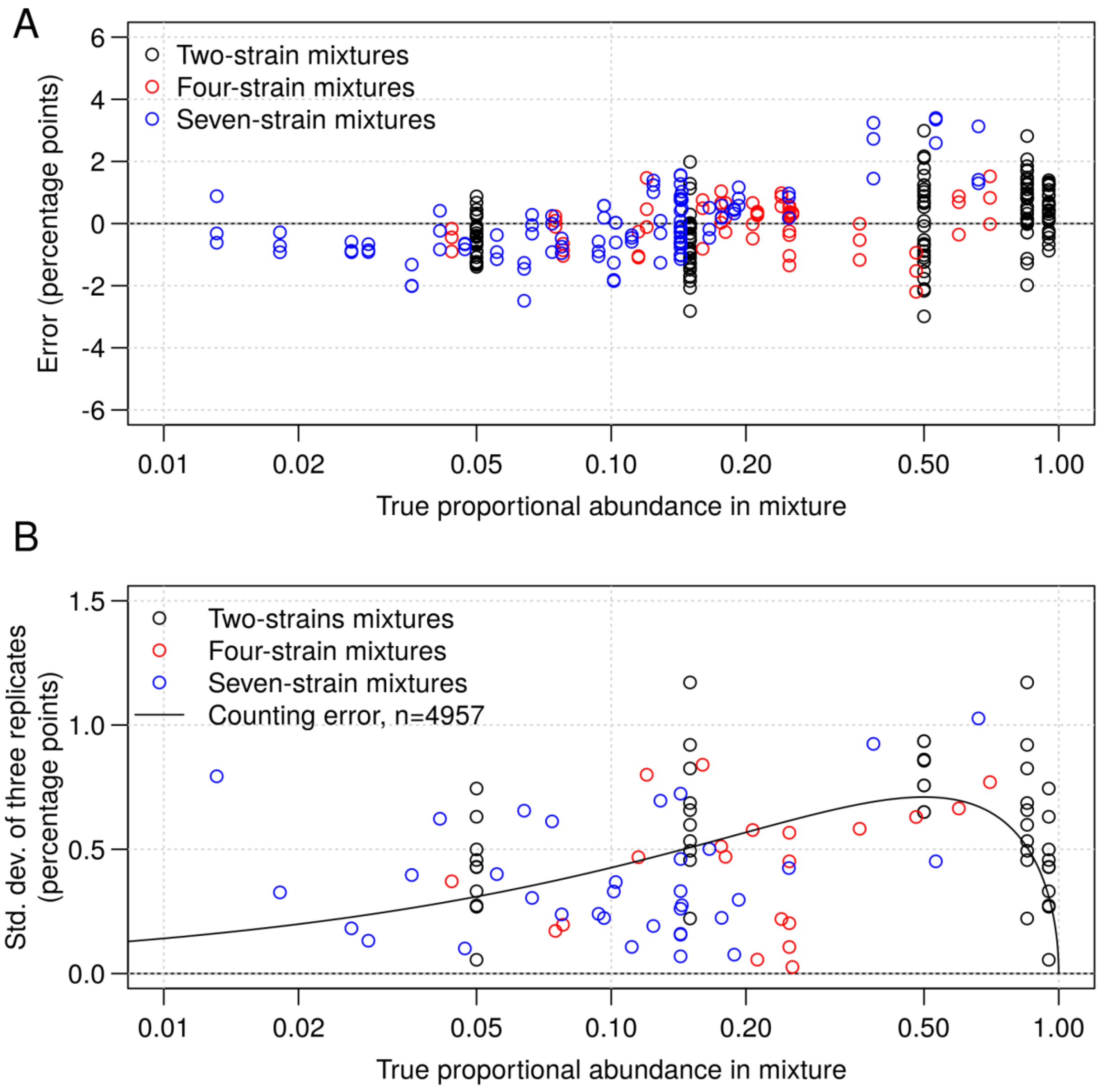

Fig S5. CASEU error magnitude is only weakly dependent on strain abundance. Related to Figures 2 and 3.

(A) Errors are similar in magnitude regardless of a strain's abundance, though there is some bias in the seven-strain mixtures at higher proportions (blue circles).

(B) Standard deviation of abundances calculated from triplicate Sanger sequencing measurements are generally around ~0.5% and are comparable to those that would be expected from counting based methods like next-generation sequencing or plate counts with *n*=4957 counts (best fit to $\sigma_{binomial} = \sqrt{\frac{p(1-p)}{n}}$).

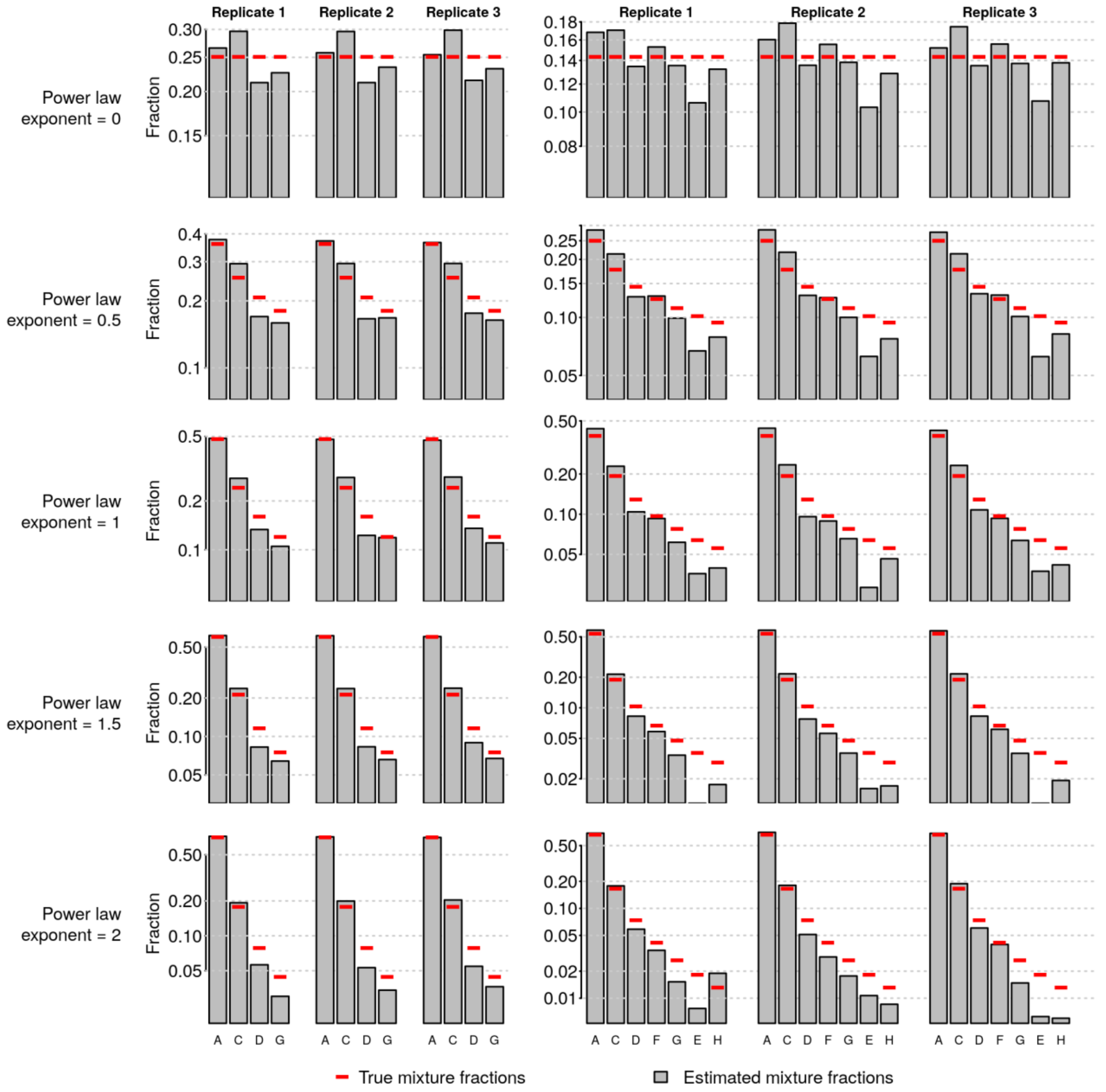

Fig. S6. Without correcting for concentration errors in stock solutions, four- and seven-strain mixtures are consistent across replicates but biased. Related to Figure 3. Data are the same as shown in Fig. 3 but without correcting for stock solution concentration errors. Solid bars are CASEU measurements after accounting for stock concentration error, whereas red lines show true mixture proportions based on power law distributions. In power law distributions, the abundance of the ith most abundant strain is proportional to $\frac{1}{i^{\alpha}}$ where α is the power law exponent.

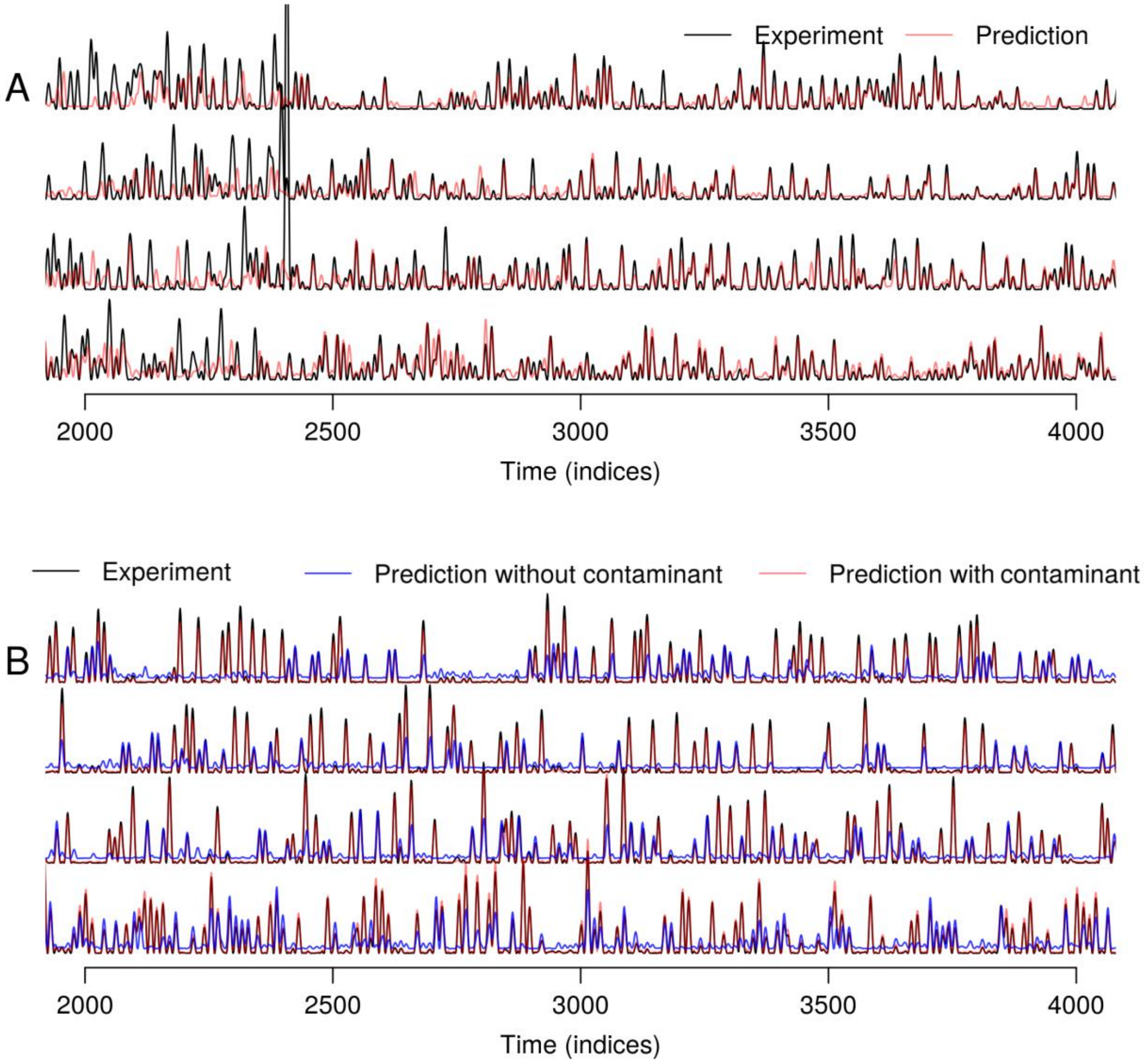

Fig S7. Assessing CASEU fits to detect sample preparation and/or sequencing errors. Related to Figure 5.

(A) The CASEU fit to a sample (red lines) does not accurately reproduce the observed mixed electropherogram (black lines). Each of the four traces shows a single fluorescence channel of the electropherogram. The best fit remained poor even when excluding the spike around t=2400.

(B) The CASEU fit to a contaminated community (blue) did not accurately reproduce the observed mixed electropherogram (black). After including the contaminating strain (identified by Illumina sequencing), the CASEU fit (red) reproduces the observed electropherogram.

E) Histogram of correlations between experiment and prediction for the model communities (n=39), showing two clear outliers (the communities shown in (A) and (C)).

```
Strain A    11    NNNNNNNNTANNNNTGNNNGTCGAGCGGAACGACAACATTGAATCTTCG
Strain B    11    GNNNNNNNTANNCNTGCAG-TCGAGCGGAACGACACTAACAATCCTTCG
Strain C    11    NNGGCNNNNACACATGCAG-TCGAGCGGAACGAGAATAG-----CTT--
                   *   *** *    **    ************* *  *      ***

Strain A    61    GAGGATTTGTTGGGCGTCGAGCGGCGGACGGGTGAGTAATGCCTAGGAA
Strain B    61    GGTGCGTTAATGGGCGTCGAGCGGCGGACGGGTGAGTAATGCCTAGGAA
Strain C    61    -----GCTATTCGGCGTCGAGCGGCGGACGGGTGAGTAATGCCTGGGAA
                         *  * ******************************** ****
```

Table S1. Strain C possesses a deletion near the beginning of the gene relative to strains A and B. Related to Figure 2. Truncated multiple sequence alignment (using Clustal 2.1) of sequences for strains A, B and C. Outside of this region, no gaps were found in the alignment. The gap in strain C begins roughly 50 bases after the end of the sequencing primer (27F) and leads to an offset of 12 bases for the remainder of the electropherogram.
